# Supplementary material for: An internal thioester in a pathogen surface protein mediates covalent host binding
Source: eLife. 2015 Jun 2;4:e06638. doi: 10.7554/eLife.06638 (PMC4450167; doi:10.7554/eLife.06638)
Supplement: Supplementary file 2. — pdf file (table). Oligonucleotide primers used in constructing expression vectors and mutants. DOI: http://dx.doi.org/10.7554/eLife.06638.024 [file elife06638s002.pdf]

Protein	Uniprot Code	Residues	Vector	Primer Sequence (5'-3')*
**Cloning**				
SfbI-A40-TED	Q1JDZ6	Asp63-Pro270	pDEST	gcccatggATGAGAAGACTGTGCCG<br>gcggatccCTAAGGTACATATTCAGCACTTAAAAG
			pOPIN-F	aggttctgtttcagggcccgGATGAGAAGACTGTGCCG<br>atggtctagaaagctttaAGGTACATATTCAGCACTTAAAAG
			pDEST-iPT	gcccatggATGAGAAGACTGTGCCG<br>gcggatccAGGTACATATTCAGCACTTAAAAG
SfbI-A346-TED	Q711F8	Asp51-Pro260	pOPIN-E	aggagatataccatgGATGAGAAGACTGTGCCG<br>gtgatggtgatgtttTGGTACAAACTCAGCACTTAAAAG
			pDEST-iPT	gcccatggATGAGAAGACTGTGCCGAATTTTAAAAG<br>gcggatccTGGTACAAACTCAGCACTTAAAAG
SfbI-A20-TED	Q01924	Asp51-Pro262	pDEST	cgccgcccatggGATGAGAAGAC<br>ccgccgggatccTTATGGTACAAACTC
			pOPIN-E	aggagatataccatgGATGAGAAGACTGTGCCTCATAG<br>gtgatggtgatgtttTGGTACAAACTCAGCACTTAGAAG
			pDEST-iPT	gcccatggATGAGAAGACTGTGCCTCATAGAGTTAG<br>gcggatccTGGTACAAACTCAGCACTTAGAAG
GfbA-TED	Q6EWI8	Asp51-Pro256	pDEST	cgccgcccatggATGAGAAGACAG<br>ccgccgggatccTTATGGTACAAACTC
FbaB-TED	Q8G9G1	Gly35-Pro241	pDEST-iPT	gcccatgGGACATGCGGAAACAAGAAATGGAGC<br>gcggatccAGGCTCTACACTAATTACTGCCTG
		Ser56-Pro241	pOPIN-E	aggagatataccatgAGTCAAGAAGAATATAATTATGAAGTTTATG<br>gtgatggtgatgtttAGGCTCTACACTAATTACTGCCTG
			pOPIN-F	aagttctgtttcagggcccgAGTCAAGAAGAATATAATTATGAAGTTTATG<br>atggtctagaaagctttaAGGCTCTACACTAATTACTGCCTG
Cpa-TED2	Q8GRA2	Ser390-Pro584	pOPIN-F	aagttctgtttcagggcccgTCAGTAGAAGCATATAATGATTTTG<br>atggtctagaaagctttaTGGATGCCACTGAGTTC
CpTIE-TED	B1R775	Ser92-Pro277	pOPIN-F	aagttctgtttcagggcccgTCTCCGACGGGTGGTC<br>atggtctagaaagctttaCGGCGTCACGAATTTG
CodTIE-TED	H2G8R6	Ser110-Pro327	pOPIN-F	aagttctgtttcagggcccgTCGGAAGACTCTAAACCGC<br>atggtctagaaagctttaCGGATTGGTGCGG
PnTIE-TED	A5MCJ6	Gly42-Pro258	pDEST	cgccgcccatgggggggatttccgaatg<br>ccgccgggatccCTAtggttcaattttccatc
Cd-TEP	Q17ZZ0	Glu171-Asp578	pHisTEV	cgccgcccatggAAGAAATAGTTATAGAG<br>ccgggatccCTAATCTGTTGGTAAACC
SaTIE-TED	I3GYQ5/I3HJ59/I3HKL2	Gln254-Ala514	pHisTEV	ccgcccatggCACAACAGAAATTAGATG<br>ccgggatccCTAAGCGCCTTCTAAAGTTG
BaTIE-TED	C3PDP7	Glu35-Glu288	pHisTEV	cgccgcccatggAAGTAATGAACAGGG<br>ccgccgggatccCTGATTCCCAATTTAC
sIPD-GFP	P42212	Ser2-Lys238	pDEST	gcccatggttatggtgAGCAAGGGCGAGG<br>gcggatccCTTGTACAGCTCGTCCATGCC
**Mutagenesis**				
SfbI-A40-TED		Cys109Ala	pDEST	GGAGTATCAAGCGTATgcTTTTAACCTAACAAAATAC<br>GTATTTTGTTAGGTTAAAAgcATACGCTTGATACTCC
SfbI-A346-TED		Cys103Ala	pDEST	CTACCAAGTTTATgccTTTAATATTC<br>GAATATTAAAggcATAAACTTGGTAG
SfbI-A20-TED		Cys97Ala	pDEST	CATATCAGGCTTATgctTTTAATTTAAAGAG<br>CTCTTTAAATTAAAagcATAAGCCTGATATG
FbaB-TED		Cys94Ala	pDEST	GTAAAACTTATCAAGGTTTTgcCTTTCAGTTAACG<br>CGTTAACTGAAAGgcAAAACCTTGATAAGTTTTAC
PnTIE-TED		Cys94Ala	pHisTEV	CTCTGATAATTTTGCCTTTgcTTTAGCGAATGGAAAAAGG<br>CCTTTTTCCATTCGCTAAAgcAAAGGCAAAATTATCAGAG
FbaB		Trp248Stop	pDEST	CGAAAGCCTTCCTtgaACATCGTTGAAGCC<br>GGCTTCAACGATGTtcaAGGAAGGCTTTCG

**Vector Construction**			
pDEST-iPT	iPT insertion	pDEST	GTTTGTACCAgcgcatattgtgatggtggatgcgtataaaTAGGGATCCGAATTCGAG<br>cgcTGGTACAAACTCAGCACTTAAAAGGTGTTGAATTGAAGTATC
	Trypsin site insertion	pDEST	GGATATCGGATCCcgtGCGCATATTGTGATGGTGGATGCG<br>acgGGATCCGATATCCGCCATGGCGCCCTG
	BamHI site insertion	pDEST	GCCATGGCGgatatcggatccgcgCATATTGTGATGGTGGATGCG<br>atcCGCCATGGCGCCCTGAAAATACAGGTTTTCGG
pDEST sIPD	N-terminal truncation	pDEST	CGGATCCGATAGTGCTACCCATATTAAATTCTCAAAACG<br>GCACTATCGGATCCGATATCAACCATGGCGC
	C-terminal truncation	pDEST	CTGTAAATGGCAAAGCAACTtAAGGTGACGCTC<br>GAGCGTCACCTTaAGTTGCTTTGCCATTTACAG
	N-terminal Cys	pDEST	CATCACCATCACGATTgCGACATCCCAACGACC<br>GGTCGTTGGGATGTCGcAATCGTGATGGTGATG
	BamHI site Insertion	pDEST	CGCCATGGTTgatatcggatccgttgataccttATCAGGTTTATCAAGTGAG<br>atcAACCATGGCGCCCTGAAAATACAGGTTTTCGGTCGT
**Lactococcus Constructs**			
SfbI-A40		pOri23	gggggatccTAACGTGGTAAGCTCATATATGTTT<br>cccgtcgacTTATCCACTATTCAGCATATTTGC
SfbI-A40:Cys109Ala	Cys109Ala	pOri23	AGTATCAAGCGTATgctTTTAACCTAAC<br>GTTAGGTTAAAagcATACGCTTGATACT

*Lowercase letters indicate bases added to facilitate cloning or that were changed for mutagenesis. Restriction endonuclease sites used for cloning are underlined.
